# Rationale of the Action of Cocaine

**Published:** 1885-07

**Authors:** 


### RATIONALE OF ANÆSTHESIA BY COCAINE.

This drug, hailed as a boon by practitioners of every branch of medical science, has been used, we may say, empyrically, as an anæsthetic. We know that it will and does produce insensibility to pain, and that its application is attended with a blanching of the surface; but it seems no one has heretofore (until very recently) attempted to explain *how* it does so—the *modus operandi*—whether its action be physiological or chemical.

It would seem that the application of cocaine was made to General Grant's throat for its analgesiant effects; and whether intentional or not, the impression was made on the mind of the public, that the physicians in charge of this illustrious patient had little or no hope of being able to *cure* him. It cannot be truthfully said that the attending physicians ever expressed in so many words, a hopeless prognosis; but the bulletins were always so worded as to leave the impression that the case was regarded as necessarily fatal in its tendency, and that no *curative* effect was anticipated from the topical use of cocaine.

We have heard the remark made, more than a score of times, that "General Grant's apparent great improvement had given the lie to the whole science of medicine;" and we heard one medical enthusiast say that "it is in very bad taste in General Grant to recover; it is a poor return for the kindness and attention shown him by the Great of the medical profession; that according to all rule and precedent, and to be consistent, and to have any regard for the professional opinion or reputation of his attendants, he ought to die; he is honor-bound to die; he is thoroughly committed to dying, and that too without delay." Readers may remember a statement which went the rounds of the medical press to the effect that "statistics show that cancer of the mouth is fatal, without operation, in ten months, and with operation, in sixteen months."

That General Grant's death was daily and hourly expected, (along about Easter,) the newspapers of the land will bear abundant testimony. We, in reading them, were always struck with the inconsistency of such prognostications, seeing at the time, his pulse and temperature were reported normal; and frequently the same telegram that would bear the sad tidings that he was "not expected to live through the night," would say perhaps, that he was up and dressed,and walked with ease into the adjoining room, or down stairs!

But we are off the point; to return.

It would seem that cocaine was applied to General Grant's throat *to relieve the pain, simply*; and no matter *how* it did it, so it was done, no one stopped to inquire.

Dr. A. F. Sampson, of Galveston, views this matter through pretty much the same smoked glasses we are now using. For instance, he says that the Doctors in charge ought to be able to give, and the country demands that they should give, a rational explanation of how the patient happened to (unexpectedly to the laity) apparently get so much better, in fact, promise to recover; they should reconcile this fact with the gloomy prognosis—and account for it in some way—and thus save the credit of the science of medicine.

Holding those views, Dr. Sampson informs us he addressed a letter to Dr. Shrady, the editor of the *New York Medical Record*, and one of the attending physicians, and suggested that the cocaine *cured* the epithelioma. This, at first glance, looks like a startling proposition, but let us see if there is not a considerable degree of soundness in it. In his letter, the writer pointed out, he says, that the action of cocaine, when applied to a diseased tissue, is physiological, and not chemical; and that the application *produces vaso-motor spasm of the terminal nerve filaments, and thus empties the engorged capillaries of their turgescence, cutting off nerve and blood supply, and starving the new growth.* (The Doctor's letter, though written sixty days ago, has never been published in the *Record*, nor noticed in the columns so far as we are aware.)

When we reflect that precisely this same effect is had upon, say uterine fibroids, and similar structures, by ergot, administered hypodermically or otherwise (except, perhaps, it has no analgesiant effect) this theory of Dr. Sampson's takes on the proportions, and assumes the aspect of a strong probability. At any rate, the suggestion does credit to Dr. Sampson, and we claim for him, and for Texas, priority in advancing such hypothesis. So far as we have been able to gather from the literature on the subject, he is the first to offer any rational and scientific explanation of the action of this wonderful drug.

Since the date of the letter above referred to, Dr. Sampson has read a paper before the *Galveston Medical Club*, embodying his views on the action of cocaine, and suggesting wider range of applicability for it; and the paper was contributed to the *Texas Courier-Record*, but owing to the crowded columns for June, only part of the valuable article was published. Dr. S. was warmly congratulated by the Club, and we are informed that a member said that he was the first to take cocaine from the domain of empiricism, and to place it on a clear and scientific basis.

